# Lights Out: Examining Sleep in Children with Vision Impairment

**DOI:** 10.3390/brainsci11040421

**Published:** 2021-03-26

**Authors:** Jessica Hayton, Jessica Marshall, Dagmara Dimitriou

**Affiliations:** 1 Department of Psychology and Human Development, UCL-Institute of Education, London WC1H 0AA, UK; jm795@sussex.ac.uk; 2 Sleep Education and Research Laboratory, UCL-Institute of Education, London WC1H 0AA, UK

**Keywords:** sleep, vision impairment, paediatric, habilitation, disordered sleep

## Abstract

Sleep is crucial for development across cognitive, physical, and social-emotional domains. Sleep quality and quantity impact domains of daytime functioning, attainment, and global development. Previous work has explored sleep profiles in typically developing children and children with developmental disorders such as Down syndrome and Williams Syndrome, yet there is a complete absence of published work regarding the sleep profiles of children with vision impairment aged 4–11 years. This is the first known study that examines the sleep profiles in children with vision impairment (*n* = 58) in comparison to 58 typically developing children (aged 4–11 years) in the UK. Sleep was measured using the Childhood Sleep Habits Questionnaire (CSHQ; parental report), actigraphy and sleep diaries. Results showed group differences in subjective CSHQ scores but not objective actigraphy measures. Surprisingly, the findings revealed disordered sleep (namely, poor sleep quantity) in both groups. Discordance between CSHQ and actigraphy measures could represent heightened awareness of sleeping problems in parents/caregivers of children with vision impairment. The implications of this study extend beyond group comparison, examining disordered sleep in ‘typically developing’ children, exploring the potential role of light perception and the importance of sleep quality and quantity in both groups.

## 1. Introduction

Sleep is a fundamental part of development, particularly pertinent in childhood. Research has established the importance of sleep in children relative to biological [1], neurological [2], cognitive [3] and behavioural functioning [4], academic attainment [5,6] and memory consolidation [7]. The benefits of good sleep quality and optimal quantity of sleep include, but are not exclusive to, more effective learning and better physical and mental health [8]. The recommended sleep duration for primary school-aged children is 9–11 h per night [9]. However, recent research suggests that poor sleep quantity and quality is a global issue [10,11]. It is estimated that approximately 20–25% of the general paediatric population develops sleep problems during childhood and adolescence [12,13].

Insufficient sleep duration and poor sleep quality (characterised by frequent night wakings) have been associated with lower performance in cognitive tasks [14,15,16] and language measures [17]. Furthermore, reduced attention [4], poor executive functioning [18] and challenging behaviours [19] have been reported. Sleep problems have also been associated with behavioural problems such as aggression, non-compliance, and impulsivity [20,21]. Physical illnesses have been shown as a contributing factor for sleep disturbances, including allergies [22], asthma and infection [23]. Given that vision impairment can have associated co-morbidities including attentional, behavioural and physical health issues [24], examining the relationship between sleep and vision impairment is considered here as necessary to further our understanding about sleep characteristics in school-aged children with vision impairment.

Vision Impairment is defined as an optical condition that cannot be corrected through prescription lenses or medical intervention [25]. Vision impairment is an umbrella term for a range of optical conditions ranging from congenital vision impairment to cerebral vision impairment, manifesting in partial sightedness (i.e., some residual vision in one or both eyes) to blindness. Children diagnosed with vision impairment have limited or denied access to observational learning, dependent on the severity and classification of the diagnosis. For children who have had vision impairment since birth/early infancy, habilitation techniques are used to support their orientation, mobility, and independent living skills as they transition into adulthood [26,27]. Despite emerging research in the field of habilitation and developing support strategies for independent living skills in areas such as dressing [28,29], consideration of sleep (or the sleep routine) as an independent living skill has been overlooked.

The examination of sleep patterns and sleep-related problems in children with vision impairment is particularly pertinent and timely. Anecdotal reports from habilitation specialists and parents of children with vision impairment have indicated several sleep issues, particularly relating to bedtime resistance (not wanting to go to bed) and anxieties surrounding bedtime. However, no substantial empirical work regarding sleep disturbance has been examined in children with vision impairment. As sleep plays such an important role in physiological, psychological and behavioural development and daytime functioning [30], the presence of a vision impairment may contribute to, or further exacerbate sleeping problems. This has been reported in other developmental disorders where vision impairment can be a characteristic but not the primary diagnosis (e.g., Williams Syndrome and Down Syndrome) [31,32,33]. Little research examines sleep and sleep-related problems in children with vision impairment, and, to the authors’ knowledge, no research has been conducted in children aged 4–11 years.

Sleep was examined in a group of 25 people with vision impairment aged 12–20 years [34] to determine if type of vision impairment (i.e., optic nerve disease) affected sleep, but categorised all participants as “young subjects”. This meant that the findings were not relative to chronological age or developmental stage. Thus far, the examined literature revealed a focus on the effects of sleep in adults with vision impairment. Findings have shown that people with vision impairment can experience suboptimal sleep [35,36,37,38]. Ocular light exposure is considered to regulate the circadian rhythm, so individuals without light perception may experience a dyssynchronous circadian rhythm, arguably resulting in poor sleep and daytime dysfunction [34,39]. Further, daytime activity was a predictor of sleep issues in adults diagnosed with retinitis pigmentosa [38] and, more generally, adults with vision impairment revealed sleep problems such as sleep latency (how long it takes to fall asleep), sleep quantity (time from falling asleep to waking up minus night waking), fragmented sleep (regular night waking) and excessive daytime sleepiness [37].

Although studies examining sleeping profiles in adults with vision impairment can be revealing, it is important to understand the developmental course of the sleep profile and its impact on other domains in vision impairment. This means that studies conducted in adult populations cannot and should not necessarily be translated onto the paediatric population of children with vision impairment. Adults who have acquired vision impairment in (early) adulthood have had prior visual experiences that could potentially have bearing on sleep quality and quantity. Therefore, the current work, examining the sleep profiles of children with vision impairment, is crucial for two reasons. First, exploration of sleep using objective and subjective measures in children (aged 4–11 years) with vision impairment (VI) has not been carried out. Thus, the current study offers preliminary insight into this overlooked field. Second, examination of sleep patterns in children with VI could also reveal an element of the function of sleep when a regulatory sensory modality (vision) is absent or compromised. To that end, the current study sought to examine the sleep profiles of children with VI aged 4–11 years and reveal similarities and differences in comparison to typically developing (TD) children of the same age range.

## 2. Materials and Methods

### 2.1. Ethics

Ethics were subject to BPS Guidelines, reviewed and fully approved by the UCL Institute of Education Staff Research Ethics committee (REC: 1137; Data Protection Registration Number Z6364106/2018/10/10/87). Parents/caregivers (as a primary contact) were informed of their rights and the rights of their child(ren) when participating in the study. This included the right to confidentiality, anonymity, and withdrawal of data. Parents/caregivers and child participants were informed about their rights to withdraw and were encouraged to ask questions throughout the study.

### 2.2. Recruitment

The recruitment was conducted electronically, predominantly via email and social media platforms across the United Kingdom. Legal guardians of potential participants contacted the researchers to receive detailed study information (for parents/caregivers and child participants) and the questionnaire link. Individuals were informed of their rights in relation to confidentiality, anonymity and withdrawal and were encouraged to ask questions prior to providing consent. This ensured that parents/caregivers and their child(ren) were aware of all procedural elements of the study. Data were collected prior to school closures and UK national lockdown resultant from the global pandemic.

Inclusion criteria were: good command of the English language, VI/VI+ conditions were identified as any optical condition that could not be corrected with prescribed glasses or lenses, + conditions were identified as any comorbidity/additional diagnosis of special educational need and/or disability which included vision impairment in multiple sites (e.g., retina and optic nerve). TD children were included on the basis that they were not under investigation for any special educational need and/or disability. All child participants were reported by parents to have normal to corrected-normal vision and hearing. 

### 2.3. Participants

In total, parents/caregivers of 142 children responded to the recruitment call for participation by approaching the research team. Of those recruited children, 18.3% dropped out of the study (*n =* 17 participants with VI due to parent/caregiver report of illness; *n =* 9 typically developing (TD)). Subsequently, the sample for analysis was N = 116 (*n =* 58 VI; *n =* 58 TD; female *n* = 56; age range 4; 07–11; 11 years; M = 8; 03 years) children from across the UK. Medical History questionnaire, devised in the researcher’s institution (*name removed for blind review*), was used for detailed characteristics of each child participant (including TD children and cause of VI) and each child’s current health, diet and lifestyle habits. The questionnaire was completed by the parent/caregiver. Table 1 shows the characteristics of the final sample. Cognitive measures were used to provide group characteristics and included sub-tests from WISC-IV Digit Span Backward and Forward [40] and Semantic Verbal Fluency and Phonemic Verbal Fluency [41]. Verbal fluency and digit span measures were not predictive of sleep performance, thus data were excluded from analysis. Classification of VI was in accordance with WHO guidelines [42].

### 2.4. Measures

Digit Span: a measurement of declarative and working memory. Considered one of few standardised measures suitable for VI populations as it does not rely upon vision to complete. The test determines an individual’s declarative and working memory based on an auditory stimulus and the child’s ability to relay/recall the number lines (increasing in number from 2 digits to 10) back to the administrator, either forwards (declarative) or backwards (working memory).

Semantic and Phonemic Verbal Fluency: The semantic verbal fluency test [41] required each participant to recall as many animals as they could as fast as possible. Scoring of semantic verbal fluency was based on the number of acceptable words for the category. The phonemic verbal fluency test required the participant to orally produce as many words as possible in one minute, beginning with a particular letter (e.g., /f/, /a/ and /s/). Scoring of the phonemic verbal fluency test relates to the amount of acceptable words for each letter.

The Childhood Sleep Habits Questionnaire (CSHQ; 52 items [43]) was used to profile children’s sleep, albeit subjectively, from the parental perspective. The CSHQ demonstrates good test–retest reliability, yielding a total sleep disturbance score in addition to eight subscales; bedtime resistance, sleep onset delay, sleep duration, sleep anxiety, night wakings, parasomnias, sleep disordered breathing and daytime sleepiness. The clinical cut off for the CSHQ is a score of ≥42. Upon completion of the online questionnaires, parents were encouraged to contact the researchers to schedule a telephone call. This was to address any questions and for each child to complete digit span and verbal fluency tasks.

Actigraphy (MotionWatch 8; CamNTech, Cambridge, UK) and sleep diaries, were used as objective sleep measures. The actigraphy device is non-invasive, worn on the non-dominant wrist of the child. For children who were ambidextrous, the watch was worn on the wrist that the child typically used less, as determined by the parent/caregiver. Data were recorded in 60 s epochs and analysed using MotionWare 1.2.28 software. Of the actigraphy data yielded, seven measures were considered; time in bed (total), assumed sleep (the time from sleep onset to offset), actual sleep (assumed sleep minus any moments of wake, expressed in time (seconds) and as a %), latency (time taken to fall asleep after “lights-out”), efficiency (% time spent asleep from onset to offset), sleep bouts (periods of wake during the night) and the fragmentation index (measure of sleep quality based on restlessness; zero represents “good sleep”, and the higher the number, the poorer the quality of sleep). Participants were requested to wear the actigraphy watch for the minimum of 5 continuous weeknights in total.

Sleep Diary: a paper-based sleep diary was used to record bed- and wake-time, night wakings and how the child felt upon awakening in the morning. Where possible, children were encouraged to keep their own paper-based or audio diary, but parents/caregivers were informed that they could maintain the diary if necessary (most parents/caregivers kept the diary). Sleep diaries were used to support actigraphy analysis.

### 2.5. Procedure

Full details of the study (for parent/caregiver and children), consent forms (for parent/caregiver and child participants) and questionnaires were available online using Qualtrics (web-based survey tool). The information and consent preluded involvement and progression in the project. Testing was conducted over one seasonal period (Greenwich Mean Time, UK) to avoid conflation of findings resultant from time difference, specifically the transition to British Summer Time. Parents/caregivers were asked to first complete the online consent (for the whole study, including actigraphy) and questionnaires (medical history and childhood sleep habits). Once completed, contact was made (initiated either by the parent/caregiver or by the research team) to complete the digit span and verbal fluency measures with the child/ren.

Verbal fluency and digit span tests were conducted over the telephone. This allowed more open recruitment as it did not rely on face-to-face meetings. Prior to the verbal and digit span measures, parents/caregivers were informed that each measure was to gather background baseline information about each child participant. If multiple participants (i.e., siblings) were in the same home, parents/caregivers ensured that only one child was present for each test. The measures were run at a mutually agreeable time for parents/caregivers/children and the researchers. Arguably running the measures remotely ensured a comfortable environment for child participants and their parent/caregiver(s), appealing to ecological validity, in keeping with the naturalistic research setting.

The telephone conversation (average time of 20 min) was also used to ensure that the parents/caregivers and their children were happy to participate, and if they had any questions, this was an opportunity to have answers. During the conversation, it was verbally agreed between the child(ren), parent/caregiver and the researcher that the actigraphy watch would be sent to the home of the participant. All parties were informed that the watch would arrive in a pack containing instructions (for how to put it on), a sleep diary and a pre-paid addressed return envelope. Parents/caregivers and children were informed to remove the watch immediately if it caused discomfort. All participants completed a sleep diary, though actigraphy data were collected for 71% of the group with VI and 55% of the TD group. The reduction in samples resulted from refusal to wear the device (tactile selectivity) in the group with VI and time/resource constraints for both groups. The reduced sample of the TD group in the study is not surprising. Children with clinical diagnoses are more familiar with research, intervention, and testing, whereas TD groups are usually less familiar with research processes and adhering to procedural instructions. Further, all participants were informed of their rights to participate, including their right to withdraw, as per the ethical procedure. Table 2 (Section 3) shows the group responses to each of the measures outlined. 

## 3. Results

Data were analysed using SPSS v 25. Full demographic, CSHQ and sleep diary data were collected from all participants (*n* = 58 VI; *n* = 58 TD) (Table 1).

ANOVA and correlations were run only on the actigraphy data and accompanying CSHQ scores gathered for the project (see Table 2). 

Scores on the parent-report CSHQ which subjectively measured sleep on eight subscales and a total sleep score were analysed using One-way ANOVAs to investigate differences, if any, between the VI and TD groups. Significant values were found on the subscales of sleep onset delay, sleep duration, sleep anxiety, night wakings, parasomnias and daytime sleepiness. See Table 3.

For the analysis of actigraphy, sleep diaries were maintained as to increase accuracy for sleep latency and wake up. As can be seen in Table 4, there were no differences between the groups on any actigraphy variables.

Correlations were run to further explore the discordance between CSHQ and actigraphy measures (Table 5). All participants’ chronological age and actual sleep time (actigraphy) were not correlated, *r*(73) = −0.226, *p* = 0.055. Between-groups correlations also revealed no statistically significant values in either group.

## 4. Discussion

This study sought to examine the differences, if any, in sleeping profiles between children with VI and TD children aged 4–11 years. Prior research studies exploring sleep differences in children with developmental disability have demonstrated differences between typical and atypical groups. Previous findings employing similar mixed methodology demonstrated that children with a developmental disability are more likely to experience sleeping problems compared to the TD population (e.g., ASD [44]; Down syndrome and Williams Syndrome, e.g., [4,15]). The findings of the current study were somewhat unexpected as both groups studied, namely, VI and TD, revealed a suboptimal quantity of sleep of just over 7 h. This is over two hours less than the recommended amount of 9–11 h for children of this age [9]. Hence, it appears that based on objective actigraphy, sleep quantity was equally poor in both groups, suggesting that VI does not necessarily appear to exacerbate poor sleep. This finding, however, is particularly surprising given the consistency in which TD control groups have been credibly reported to sleep over 8 h [4].

The current TD sample appeared to have numerous sleep problems. These included a significantly large number of nocturnal wakings, short sleep of over 7 h and longer sleep latency. However, the findings in the current study are not entirely discordant with other published works relative to actigraphy performance of children with developmental conditions compared with TD controls (e.g., in autism research, see [45,46]). As the current study is the first exploring sleep in children with VI, there is an absence of comparative data to comprehensively explore this phenomenon. Fallone et al. [47] reported that excessive sleepiness in children is a growing concern, and adolescents are increasingly likely to sacrifice sleep quantity in favor of screen use [48].

Had the TD group presented with ‘typical’ sleeping behaviors relative to their chronological age and published guidelines, the findings of the current study would alter. Children with VI, in the current study, do demonstrate sleeping problems in relation to the typical norm. Comparisons between the findings of the current study with published works arguably reveal that the differences yielded in CSHQ might corroborate with the notion that the presence of VI has a negative effect on quality and quantity of sleep.

Closer examination of the actigraphy data in the current study effectively revealed two groups displaying sleep problems. The first, expected, group was that of children with VI. The second, unexpected, group was that of the TD “control” group who also presented sleeping problems relative to chronological age, quality, and quantity. Sampling may begin to explain this finding, as parents/caregivers of TD children may have participated in this project because they suspected sleep problems in their child(ren), despite this response not being intentionally primed during recruitment. This is the nature of real-world research; whereby voluntary participation may be increased by (un)conscious bias toward a subject or research theme. Therefore, parent/caregivers (of both groups) may be motivated to participate in the study to receive support or develop greater understanding of their child(ren)’s sleeping profile. Additionally, findings in the current study may relate to parental knowledge of sleep, namely, parents of children with disabilities show greater awareness of sleep hygiene [49]. Though statistical differences were not found in the current sample, the data revealed a subgroup of TD children who may also substantially benefit from clinical sleeping interventions, so in future work, detailed consideration of the “control” group ought to be explored.

The Medical History Questionnaire and follow-up correspondence asked for details surrounding prematurity, birthweight, additional diagnoses, timing of onset (of vision impairment), visual diagnosis, severity of vision impairment and visual acuity. Some parents/caregivers were unable to provide such detailed information. Reasons for this remain speculative, though it ought to be acknowledged that the parents/caregivers may not understand their child’s diagnosis and may be experiencing grief and reconciliation in understanding their child’s visual needs and diagnosis. The second potential explanation for the findings related to light perception. Except for four participants with VI, all recruited participants had light perception (albeit to varying degrees, based on clinical diagnosis). Previous work in adult populations suggested that the absence of light perception may affect sleep quality and quantity [33,38], as ocular light supports synchronicity of the circadian rhythm. In this study, 54 children with VI were reported by parents as having light perception, and the objective measure of sleep (actigraphy) did not show differences between groups, as the circadian rhythm may not have been substantially affected by the absence of light. The presence of light perception could also potentially explain the lack of difference in sleep latency in both groups. Despite the lack of group differences in the actigraphy measure, the findings are still pertinent. This is because suboptimal sleep and the objective sleep profiles are not specific to population or group. Objectively, both groups displayed insufficient sleep (relative to recommendations) but interestingly, the adverse between-groups effects of this were only revealed in the subjective CSHQ measure. 

The findings of the parental-report CSHQ unsurprisingly corroborated with anecdotal reports from parents/caregivers and Habilitation Specialists (whose reports were the basis for this exploration). The CSHQ results showed that children with VI generally had greater sleeping problems (as indicated by the total scores of problematic sleep). The differences were also apparent in seven (of eight) subdomains of the CSHQ, i.e., bedtime resistance, anxiety surrounding bedtime, night wakings, parasomnias, sleep disordered breathing, sleep onset delay and sleep duration. Group differences were not found in the CSHQ subdomain of daytime sleepiness, and potential reasons for this could be the similarity in sleep diary and actigraphy measures for both groups, meaning that both groups were equally as sleepy (or not) through the day. Of the parental-report CSHQ data, the significant differences in sleep onset delay and sleep duration were not supported by the actigraphy data, as sleep latency and actual sleep time were not substantially different. This is noteworthy, as it shows the potential discordance in subjective (CSHQ) and objective (actigraphy) sleep measures. This discordance has been reported in previous literature surrounding sleep and developmental disability, so is not a surprising finding.

The current study supports the notion that sleeping problems are not subjective to specific populations (i.e., children with VI), building upon existing evidence that sleep deprivation is a global epidemic, evidenced from 12 years old (early adolescence) into adulthood [11]. The recruited sample did not appear to sleep for the recommended amount of time relative to their chronological age, so raising awareness of this via research and reporting is essential for moving forwards. Considering sleep and the sleeping routine as a fundamental independent living skill in habilitation practice could support a consistent sleep routine, and this is also applicable to TD children. Ideally, habilitation support should be offered as early as possible (from birth/early infancy) to reap the developmental rewards through childhood into adult life [27]. As sufficient quality and quantity of sleep supports physical, cognitive, and social-emotional development, it is crucial that during these early years of development, every opportunity to support and enhance growth are seized. Further research in the field of habilitation could potentially examine the sleeping routine (up to 1 h before bed) to see if consistent and structured support may yield positive effects on total sleep time and support the developmental trajectory into adolescence and adulthood, though, conversely, the recommendations and guidelines of paediatric sleep may need revision to appropriately capture changes and advancements in society and environments. 

This is the first and largest empirical study examining sleeping profiles in children with VI aged 4–11 years. The purpose was to explore differences, if any, in terms of sleeping profiles between children with VI and children with TD. Differences were reported on subjective questionnaire measures regarding sleeping profiles of children with VI. This was not corroborated by objective actigraphy measures, suggesting discordance between subjective reporting and objective measurement. The actigraphy data revealed that both groups had short sleep duration in comparison to the paediatric recommendations. As sleep is an essential independence skill that has bearing on cognition, learning and daytime functioning, these findings ought not to be ignored. Poor sleep quality and quantity is a public health concern of global proportion. The current findings show that parents of children with developmental conditions such as VI may have better awareness of problematic sleep unlike parents of typically developing children; hence, gaining better sleep ought to be considered for all children from an education, health, and social care perspective.

Future research in this field would benefit from comparison between children with and without light perception. Biomarkers such as cortisol and melatonin could also be measured as a predictor of sleep quality and potential disturbances to circadian rhythm that have not been captured in the present study. Also, parents of young children play a vital role in optimal sleep hygiene, hence, improving parental sleep knowledge in particularly about the sleep duration requirements of children may result in positive outcomes.

## Figures and Tables

**Table 1:** Characteristics of the sample (*n* = 116).

Demographic Characteristic	VI *n* = 58	TD *n* = 58
Chronological age in months		
Mean (SD)	98.40 (24.03)	102.83 (24.31)
Sex (%)		
Male	31 (53.5%)	29 (50%)
Female	27 (46.5%)	29 (50%)
Cognitive Measures M (SD)		
Valid *n* (%)	45 (77.6%)	29 (50%)
Semantic Verbal Fluency (raw score)	14.04 (5.70)	15.31 (6.01)
Phonemic Verbal Fluency (raw score)	16.49 (10.47)	18.97 (8.21)
Digit Span Forward (raw score)	7.27 (2.13)	8.55 (2.08)
Digit Span Backward (raw score)	5.58 (1.85)	6.10 (2.09)
Timing of vision impairment onset (%)		
Birth	38 (65%)	-
Early ≤7 months	2 (4%)	-
Late (>2 years) 2.5 years–4 years	3 (5%)	-
Not recorded	15 (26%)	-
Severity of Vision Impairment *		
Moderate Vision Impairment	9 (16%)	-
Severe Vision Impairment	7 (12%)	-
Blindness	6 (10%)	-
Not Classified	36 (62%)	-
Diagnosis by site of vision impairment ^†^		
Whole globe and anterior segment	15 (26%)	-
Lens; cataract	2 (4%)	-
Retina	10 (17%)	-
Optic Nerve	9 (16%)	-
Cerebral or visual pathways	13 (22%)	-
Ideopathic nystagmus	23 (40%)	-
High refractive error	3 (5%)	-
Not Classified	15 (26%)	-
No Light Perception ^§^	4 (7%)	-
Additional need/s diagnosis ^§^	15 (26%)	-

* Severity of vision impairment using WHO classification of vision impairment see [41]; moderate VI refers to visual acuity of 0.6–1.0 logMAR 6/24–6/60 Snellen; severe VI refers to visual acuity of 1.1–1.3 logMAR or 5/60–3/60 Snellen; blindness refers to 1.4 logMAR or 2/60 Snellen; not classified refers to participants with a diagnosis of VI, though acuity data were not provided/known by parent/caregiver. ^†^ Diagnosis by site does not equate to 100% as some children had diagnoses in multiple sites. ^§^ No light perception and additional diagnosis were based on parent/caregiver report. VI, vision impairment, TD, typically developing.

**Table 2:** Response rate to measures according to group.

	Measure				
	MHQ *	CHSQ *	Standardized Measures of Cognitive Functioning	Sleep Diary	Actigraphy ^†^
VI (*n* = 58)	58 (100%)	58 (100%)	45 (78%)	58 (100%)	41 (71%)
TD (*n* = 58)	40 (69%)	58 (100%)	29 (50%)	58 (100%)	32 (55%)

* MHQ refers to the parental-report medical history questionnaire; CSHQ refers to the long version of the Childhood Sleep Habits Questionnaire; both measures were completed by the legal parent/guardian ^†^ Actigraphy was measured using watches worn by child participants.

**Table 3:** Group mean scores, standard deviations, and group differences in CSHQ (ANOVA).

Subscales					
	VI (*n* = 58)	TD (*n* = 58)	F	*p*	R^2^
Bedtime Resistance	8.09 (2.81)	7.21 (1.93)	3.87	0.052	0.03
Sleep Onset Delay	1.90 (.85)	1.36 (.667)	14.07	<0.001	0.11
Sleep Duration	5.25 (2.05)	3.59 (1.11)	29.48	<0.001	0.21
Sleep Anxiety	6.65 (2.05)	5.38 (2.05)	11.12	<0.001	0.08
Night Wakings	5.05 (1.91)	3.90 (1.27)	14.68	<0.001	0.11
Parasomnias	9.63 (2.92)	8.43 (1.66)	7.39	0.008	0.06
Sleep Disordered Breathing	3.58 (1.17)	3.22(.750)	3.78	0.054	0.03
Daytime Sleepiness	12.84 (3.96)	10.60(3.24)	11.08	<0.001	0.08
Total Score	49.89 (9.15)	39.95 (7.13)	42.61	<0.001	0.27

**Table 4:** Brief demographics mean scores (SD) and group differences using ANOVA for actigraphy variables.

	VI (*n* = 41)	TD (*n* = 32)	F	*p*	η_p_^2^
Chronological Age (Months) M (SD)	100.56 (24.8)	100.81 (27.8)	-	-	-
Sex (%)			-	-	-
Female	22 (53.7%)	16 (50%)			
Male	19 (46.3%)	16 (50%)			
Bedtime ((hh:mm)	20:19 (1:09)	20:35 (0:47)	1.289	0.261	0.018
Wake Time (hh:mm)	06:54 (0:47)	7:07 (0:33)	1.664	0.201	0.023
Time in Bed (hh:mm)	10:45 (01:08)	10:31 (00:47)	1.045	0.310	0.015
Assumed Sleep (hh:mm)	9:55 (1:16)	9:41 (0:36)	0.905	0.345	0.013
Actual Sleep Time (hh:mm)	7:22 (1:37)	7:19 (0:35)	0.036	0.850	0.001
Actual Sleep %	76.08 (7.5)	75.6 (4.6)	0.079	0.780	0.001
Sleep Efficiency (%)	70.0 (9.8)	69.9 (5.9)	0.042	0.838	0.001
Sleep Latency (hh:mm)	38 (0:28)	37 (0:27)	0.034	0.853	0.001
Mean Sleep bout time (mm:ss)	10:24 (06:49)	10:06 (04:41)	0.048	0.828	0.001
Fragmentation index	36.6 (13.1)	34.7 (10.01)	0.417	0.520	0.006

**Table 5:** Correlation between objective actigraphy measure parent-report CSHQ total.

	VI (*n* = 41)			TD (*n* = 32)		
	*n*	r	*p*	*n*	r	*p*
Actual sleep vs. sleep duration	41	−0.150	0.350	32	−0.164	0.369
Sleep latency vs. sleep onset delay	41	0.082	0.612	32	0.172	0.348
Sleep bouts vs. night wakings	41	0.050	0.758	32	0.296	0.100

## Data Availability

The data are not publicly available due to the ethical approval stating only processed data will be made available and to preserve the anonymity and confidentiality of the participants.
